# The Use of *Galleria mellonella* Larvae to Identify Novel Antimicrobial Agents against Fungal Species of Medical Interest

**DOI:** 10.3390/jof4030113

**Published:** 2018-09-19

**Authors:** Kevin Kavanagh, Gerard Sheehan

**Affiliations:** Medical Mycology Laboratory, Department of Biology, Maynooth University, Maynooth, Co. Kildare W23F2H6, Ireland; gerard.sheehan.2103@mumail.com

**Keywords:** Galleria, model organism, antifungal, in vivo testing, immunomodulatory

## Abstract

The immune system of insects and the innate immune response of mammals share many similarities and, as a result, insects may be used to assess the virulence of fungal pathogens and give results similar to those from mammals. Larvae of the greater wax moth *Galleria mellonella* are widely used in this capacity and also for assessing the toxicity and in vivo efficacy of antifungal drugs. *G. mellonella* larvae are easy to use, inexpensive to purchase and house, and have none of the legal/ethical restrictions that are associated with use of mammals. Larvae may be inoculated by intra-hemocoel injection or by force-feeding. Larvae can be used to assess the in vivo toxicity of antifungal drugs using a variety of cellular, proteomic, and molecular techniques. Larvae have also been used to identify the optimum combinations of antifungal drugs for use in the treatment of recalcitrant fungal infections in mammals. The introduction of foreign material into the hemocoel of larvae can induce an immune priming effect which may operate independently with the activity of the antifungal drug. Procedures to identify this effect and limit its action are required.

## 1. Advantages of *Galleria mellonella* Larvae

The insect immune response displays many structural and functional similarities to the innate immune response of mammals [1]. For example, insect hemocytes show many similarities (e.g., phagocytosis, superoxide production) to mammalian phagocytes [2] and many of the receptors (e.g., Toll) and response pathways (e.g., coagulation and melanisation) in insects are comparable to those in mammals [1,3]. Due to the presence of these conserved features, insects are now widely used to assess the virulence of fungal pathogens and to determine the toxicity and in vivo efficacy of novel and conventional antifungal drugs and produce results comparable to those that may be obtained using mammals.

**Figure 1:**
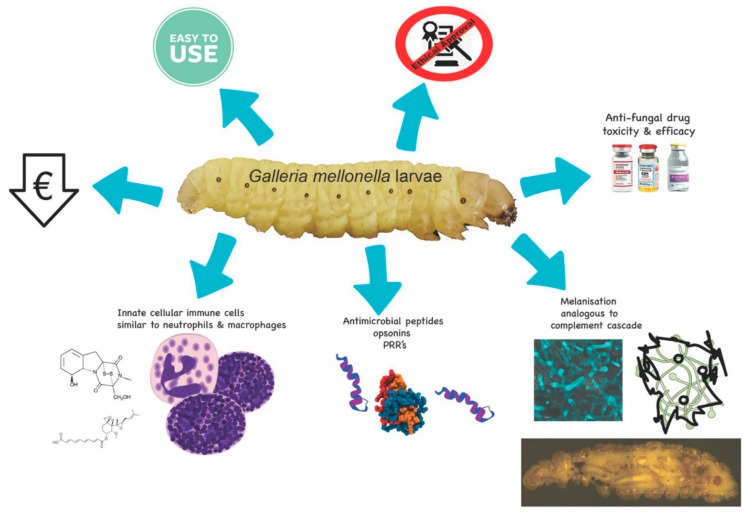
Schematic diagram of some of the advantages associated with using *G. mellonella* larvae. Larvae are inexpensive to purchase, easy to use, are not subject to the ethical or lethal restrictions associated with mammalian testing, and can be employed for testing the toxicity and efficacy of a range of novel antifungal drugs. These advantages are possible due to the similarities between the mammalian innate immune response and the insect immune system.

**Figure 2:**
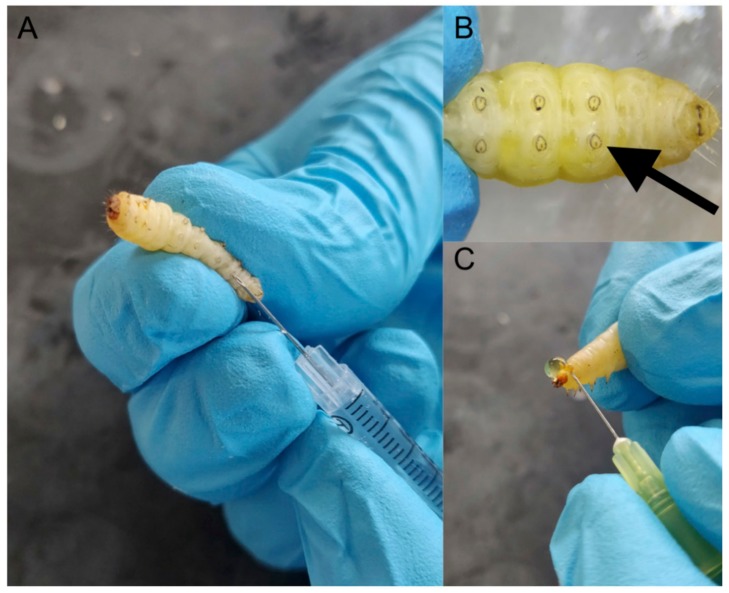
General procedures associated with using *G. mellonella* larvae. Larvae are inoculated with a specific volume (e.g., 20 μL) through the last left proleg using a syringe (**A**), magnified image of last left proleg (**B**), demonstration of the method to obtain hemolymph from larvae (**C**).

A wide range of insects is now used as in vivo models (e.g., *Drosophila melanogaster, Manduca sexta, Bombyx mori*) [4,5] but larvae of the Greater wax moth (*Galleria mellonella*) are a popular choice due to their ease of inoculation, low cost and the ability to generate results in 24–48 h [6,7] (Figure 1). The low cost of larvae means it is possible to perform many replicates and produce statistically valid results. *G. mellonella* larvae are easy to house and their use has none of the legal or ethical constraints that restrict the use of mammals. Larvae are amenable to incubation at 37 °C which means that many temperature dependent virulence factors of human pathogens are active. In addition, *G. mellonella* larvae may be easily and accurately inoculated via intra-hemocoel injection (Figure 2A,B), by force-feeding or by rolling on a layer of spores and a number of parameters may be employed to assess their response to infection. These include mortality, extent of melanization, alteration in hemocyte density and/or function, changes in microbial load, formation of pupa, movement, alteration in gene expression and variations in the proteome. Another advantage is that due to their size and ease of handling it is possible to administer a defined inoculum by force-feeding or intra-hemocoel injection to larvae. Larvae can be used for pharmacokinetic and pharmacodynamics studies and larvae produce a large volume (80–100 μL/larva) of hemolymph (Figure 2C) (analogous to mammalian blood) which can be analysed by a variety of methods [8,9,10]. Hemocytes (immune cells) can be isolated from larvae and subjected to a range of ex vivo cellular assays in response to microbial pathogens and/or to determine the effect of chemical agents [11,12,13,14]. The use of *G. mellonella* larvae, as with any model organism, has some disadvantages. For example, a lack of mutant strains and larvae may not be a suitable model for some microbial species. However many of the disadvantages detailed by Tsai et al. (2016) have been addressed in the recent past [15,16,17].

The immune responses of larvae have been recently documented at the proteomic, transcriptomic and epigenetic level due to advances in technologies such as label-free proteomics and miRNAs and the recent sequencing of the *G. mellonella* genome opens new avenues of research [16,18,19]. Although larvae do not possess an adaptive immune response associated with long-lived memory cells and antibodies, they possess immunological memory as evidenced by immune priming resulting in protection against a lethal infection if initially pre-exposed to a low level of the pathogen. The absence of an adaptive response can be an advantage as it allows the researcher to study in detail the interactions of pathogen and the innate immune response without interference from the adaptive response [20].

The response of *G. mellonella* larvae to infection with *Candida albicans* [21,22] and *Aspergillus fumigatus* [23,24,25] shows a strong correlation to the results obtained using mice (Table 1). *G. mellonella* larvae are excellent models to study the virulence of *Candida* species [26] and larvae have been utilized to develop an infection model for *Cryptococcus neoformans* [27,28]. Invertebrates such as *Acanthamoeba castellanii, Caenorhabditis elegans, Dictyostelium discoideum, D. melanogaster* and *G. mellonella,* have been employed to characterize the molecular mechanisms which *Cryptococcus neoformans* utilizes to attack the host [29]. The results revealed that several virulence-related genes previously associated with *C. neoformans* virulence in mammals also played a role in establishing infection in *G. mellonella*. *C. neoformans* serially passaged in *G. mellonella* larvae showed an enhanced ability to kill mice if administered by intra-tracheal or intravenous route, although their ability to kill *G. mellonella* larvae remained unchanged. In addition, microarray analysis showed passaged cells had increased expression of genes involved in the oxidative stress response (e.g., peroxiredoxin (Tsa1) Super oxide dismutase (Sod2) [30].

*G. mellonella* larvae can also be used to model the development of systemic Candidosis and Aspergillosis and show many pathologies also found in systemic infection in mammals. Larvae infected with *C. albicans* showed changes similar to those observed in mice challenged with the same pathogen, such as alterations in immune cell density, increased abundance of antimicrobial peptides, and in proteins indicative of tissue invasion [19]. In the case of *A. fumigatus* infected larvae there is the development of nodules containing viable fungal mycelia and hemocytes, and these are similar in composition to the fungal granulomas found in systemic *A. fumigatus* infection in mammals [37]. *A. fumigatus* conidia germinate, form hyphae at the point of inoculation, and invade through larval tissue to produce disseminated aspergillosis, and this shows similarities to the process in mice. The ability to model disease processes in larvae creates the possibility of characterizing the efficacy of antifungal therapy both in terms of larval survival but also in the reduction of the symptoms associated with infection.

## 2. Utilization of *G. mellonella* Larvae for Measuring Relative Toxicity In Vivo

Insects may be used to assess the relative toxicity of a variety of agents, including antifungal agents, and the results show a strong correlation to those obtained using mammals [38]. In one case *G. mellonella* larvae were administered, by intra-haemocoel injection or by force-feeding, a variety of food additives and the LD_50_ values were determined [38]. The values obtained showed a strong correlation to the LD_50_ values determined in rats. Administration of a wide range of compounds to larvae by intra-hemocoel injection allowed differentiation of the compounds based on relative toxicity and this correlated well with the toxicity as assessed in cell culture systems and in mammals [39]. A strong correlation between the LD_50_ values of a range of chemicals (e.g., 4-methyl umbelliferone, umbelliferone, and 7-ethoxycoumarine) in silkworm larvae (*Bombyx mori*) and in mammals was also established and similar metabolic pathways to detoxify the chemicals in both groups of animals were demonstrated [40]. 

**Figure 3:**
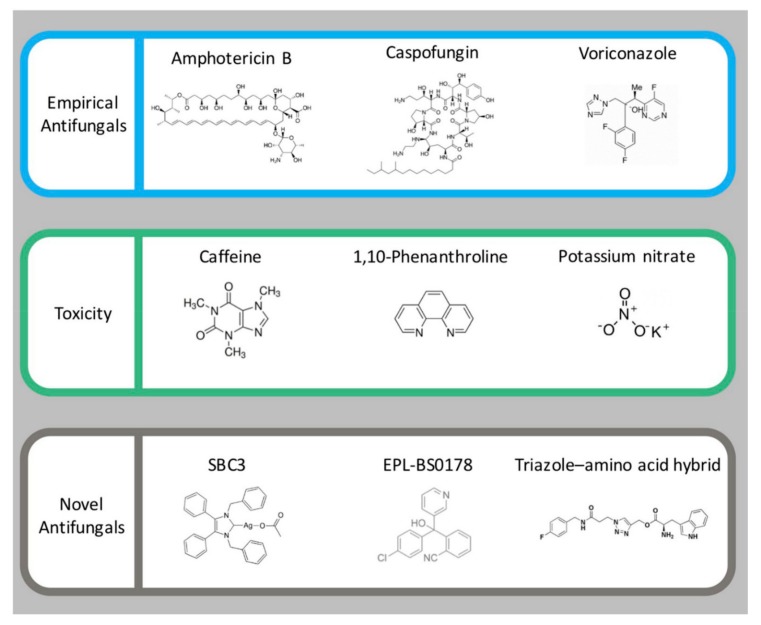
Examples of uses of *G. mellonella* larvae to assess the in vivo toxicity and efficacy of a range of antifungals and chemical agents. Structures of empirical antifungals amphotericin B [43], caspofungin [44], voriconazole [31]. Chemicals used for toxicity studies; caffeine [9], 1,10-Phenanthroline [41] and potassium nitrate [45]. Novel antifungals; SBC3 (1,3-dibenzyl-4,5-diphenyl-imidazol-2-ylidene silver(I) acetate) [46] active against *C. albicans*, EPL-BS0178 [47] active against *M. mycetomatis* and novel triazole–amino acid hybrid (1-(3-(4-fluorobenzylamino)-3-oxopropyl)-1H-1,2,3-triazol-4-yl)methyl 2-amino-3-(1Hindol-3-yl)propanoate) [48].

*G. mellonella* larvae can be employed to assess the toxicity of antimicrobial agents and results show a strong correlation to the toxicity as measured in mammals. In an assessment of the relative toxicity of 1,10 phenanthroline, 1,10 phenanthroline-5,6-dione and related copper and silver complexes a strong correlation between the response of *G. mellonella* larvae and Swiss mice was established [41] (Figure 3). While the rank order of toxicity differed slightly, the most toxic compound in larvae was also the most toxic in mice indicating the potential of the larval system for rapidly, and cost effectively identifying the toxicity of compounds prior to murine testing. All compounds were less toxic than cisplatin in larvae and mice, and also well tolerated in nude mice. Analysis of the in vivo toxicity of ionic liquids (salts containing poorly co-ordinated ions that remain in liquid form below 100 °C) in *G. mellonella* larvae revealed a strong correlation to the toxicity as assessed in other invertebrate models (*Daphnia magna* and *C. elegans*) and demonstrated that the larval system was a sensitive and reliable in vivo model system [42].

As well as being suitable to assess the relative in vivo toxicity of compounds, larvae may also be used to study the mode of action of selected compounds and show equivalent results to those found in vertebrates. Administration of potassium nitrate (Figure 3) to *G. mellonella* larvae produced a response comparable to that observed in mammals and this included an elevated hemocyte density but hemocytes showed reduced fungicidal activity [45]. Larvae administered potassium nitrate also showed alterations in proteins involved in mitochondrial function (e.g., mitochondrial aldehyde dehydrogenase), metabolism (triosephosphate isomerase) and nitrate metabolism (glutathione *S*-transferase), effects also observed in mammals exposed to this compound. Caffeine (Figure 3) administration to *G. mellonella* larvae resulted in developmental delays (e.g., reduced pupation) and lack of movement which were also observed in Zebra fish embryos [49]. Proteomic analysis of alterations in the brain of larvae administered caffeine indicated increased abundance of proteins associated with brain trauma and decreased abundance of proteins implicated in development and protein degradation [9].

A series of novel copper phenanthroline-phenazine cationic complexes which display promising chemotherapeutic potential decrease *G. mellonella* survival dependent upon enhanced nuclease activity, this is evident at the proteomic level with enrichment of metabolic and detoxification pathways. These results indicate that larvae may be used to assess the tumoricidal activity of novel anti-neoplastic agents [50].

## 3. Use of Larvae for Assessing Antifungal Activity In Vivo

### 3.1. Drug Assessment against Pathogenic Yeast

**Figure 4:**
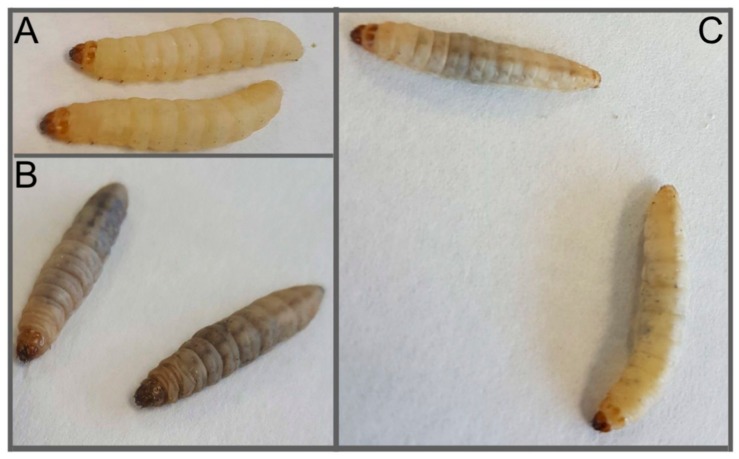
Images of *G. mellonella* larvae infected with *Candida albicans* (5 × 10^5^/larva) 24 h post-infection. (**A**) Control larvae administrated PBS (40 μL), (**B**) larvae administrated *C. albicans* 24 h post-infection, (**C**) larvae administrated *C. albicans* and amphotericin B (1 mg/kg 2 h post *C. albicans* infection) 24 h post-infection.

One of the advantages of using *G. mellonella* larvae is that the in vivo activity of novel and conventional antifungal agents can be quickly established (Table 2) and this can inform subsequent synthesis of novel derivatives or help establish relative dosage prior to use in mammals. *G. mellonella* larvae can be rescued (i.e., increased survival and decreased symptoms, e.g., melanization) from a lethal inoculum of *C. albicans* (5 × 10^5^/larva) by an intra-hemocoel dose of amphotericin B (1 mg/kg; a dose comparable to clinical dosing levels (maximum 1.5 mg/kg/day) two hours post-infection (Figure 4). Rowan et al. (2009) demonstrated increased survival of larvae inoculated with *C. albicans* and followed up 1 and 4 hours later with the novel antifungal compound Ag_2_(mal)(phen)_3_ [51]. Prophylactic administration of the antifungal agent also resulted in increased survival and experiments indicated that the agent, as well as exerting inherent antifungal activity, also stimulated the immune response of larvae as measured by increased hemocyte density in larvae and the elevated expression of the gene coding for *gallerimycin*, which has well established antifungal properties. This immune stimulation effect was also evident when larvae were administered the echinocandin antifungal drug, caspofungin (Figure 3). As well as exhibiting increased survival following *C. albicans* infection, caspofungin-treated larvae showed increased resistance to *Staphylococcus aureus* infection although the drug displays no inherent antibacterial activity [44]. Analysis revealed that administration of caspofungin to larvae resulted in increased hemocyte density and an elevation in the expression of genes coding for *IMPI* and *transferrin*. A similar immune priming effect inducing protection against *S. aureus* infection in larvae was also observed following administration of micafungin to *G. mellonella* larvae [52]. When examining the antimicrobial activity of a novel agent, hemocyte densities should be examined 24, 48 and 72 h post-introduction to control for and determine if the antifungal effect observed is true and not as a result of a stimulated immune response. It was also demonstrated that murine macrophages had enhanced fungicidal activity when pre-treated with micafungin. Prophylactic treatment of mice lead to elevated levels of a number of pro-inflammatory cytokines and enhanced phagocytic ability of neutrophils and macrophages [52].

This immune priming effect can also be induced by administration of β-glucan, a component of the fungal cell wall which displays no antifungal activity, to larvae. Following β-glucan administration larvae showed increased resistance to *C. albicans* infection and this was mediated by a dose-dependent cellular and humoral immune response [54]. Administration of high doses of glucan to larvae (e.g., 15–60 μg/larva) induced elevated hemocyte densities, reduced density of yeast cells and increased survival. Larvae also showed elevated abundance of a range of antimicrobial peptides (e.g., archaemetzincin) and immune proteins (e.g., hemolin) [54]. Administration of host derived extracellular nucleic acids to *G. mellonella* larvae lead to increased expression of antimicrobial peptides and a reduction in hemocyte density. The combined effect resulted in protection of larvae infected by *Photorhabdus luminescens* indicating that immune stimulation can help overcome a potentially lethal infection [55].

These findings are critically important to consider when using *G. mellonella* larvae, or other insect models, to assess the in vivo efficacy of antifungal drugs. As has been demonstrated the test compound may induce an immune response once introduced into the insect hemocoel. This response may target the invading pathogen and can be mediated by alterations in the hemocyte density and in the abundance of antimicrobial peptides in the insect hemolymph. It is essential to differentiate between the anti-microbial effect due to the increased immune response induced by the compound and the inherent anti-microbial activity of the agent. Interestingly a carbene silver(I) acetate complex demonstrated excellent in vivo anti-*Candida* activity but was shown not to trigger an immune response in larvae (Figure 3). It was postulated that the relatively small size of the complex was not detected by the larval immune response and thus failed to induce an immune response [46]. A group of novel triazole-amino acids hybrid compounds was assessed for their anti-*Candida* activity in vitro, and in vivo using *G. mellonella* larvae. The results showed the compounds did not provoke an immune response in larvae and that administration of compound to larvae resulted in increased survival and reduced yeast cell proliferation [48].

Gu et al. (2018) demonstrated that the combination of tetracycline and fluconazole at concentrations equivalent to therapeutic doses in humans rescued larvae from a lethal inoculum of azole resistant *C. albicans* CA10 compared to fluconazole alone and this was due to a four-fold decrease in fungal burden and fewer infection sites throughout larvae [56]. The Santos groups examined the antifungal efficacy of a range of antifungals on *Candida haemulonii* complex (*C. haemulonii, C. duobushaemulonii and C. haemulonii var. vulnera*) and non-*albicans* species, with the former demonstrating resistance to first line antifungals (e.g., fluconazole, amphotericin B) in *G. mellonella* larvae [57].

*Candida tropicalis* dose-dependently killed *G. mellonella* larvae at both 30 °C and 37 °C but larval hemocytes phagocytosed *C. tropicalis* cells slower than those of *C. neoformans*. Amphotericin B, caspofungin (2, 4 mg/kg; comparable to clinical dosing levels), fluconazole and voriconazole all produced protective effects in vivo at clinically relevant doses but amphotericin B (4 mg/kg) and fluconazole (3, 6, and 12 mg/kg) significantly decreased fungal burden and melanised nodule formation in larval tissue as evident from tissue sections [31].

The response of *G. mellonella* larvae to infection with *Candida glabrata* was studied and infection was shown to be temperature and dose-dependent and the response of the larvae to infection included melanisation and alterations in the hemocyte density. No protection from infection was achieved by administering fluconazole to larvae post-infection, although amphotericin B and caspofungin administration increased larval survival [53]. *G. mellonella* larvae have also been utilized for assessing the in vivo activity of amphotericin B, flucytosine, and fluconazole following challenge with *C. neoformans* [27]. Combination therapy consisting of pedalitin and amphotericin B act in synergy against *C. neoformans* infection to improve survival, fungal burden and histopathology in both *G. mellonella* larvae and in BALB/c mice [58].

Larvae provide a quick and convenient means to assess the potential of novel antifungal therapies prior to murine testing. *G. mellonella* larvae and mice have been employed to assess the efficacy of antimicrobial peptides in combination with caspofungin for treating *C. albicans* infections. There was strong agreement between results since treatment of infected larvae and mice with caspofungin and DsS3 (1–16) resulted in enhanced survival of larvae and mice compared to untreated controls and those that received the agents individually [59]. An evaluation of the effect of acetylcholine (ACh) on *G. mellonella* larvae indicated it enhanced the activity of hemocytes and protected larvae from *C. albicans* induced infection [60]. ACh inhibited the *C. albicans* dimorphic switch and biofilm formation and this, together with the elevated immune response, may have prevented disease development. This result indicated the potential of using ACh as either an antifungal agent with direct effect on the yeast or as an immune stimulant in mammals.

### 3.2. Drug Assessment against Filamentous Fungi

*G. mellonella* larvae have been widely used to study azole resistance in *A. fumigatus* [61]. Alaczar-Fuoli et al. (2015) demonstrated voriconazole selected for *Aspergillus lentulus* in mixed infections with *A. fumigatus*, which together respond differentially to larval hemocytes and produce distinct histological features (e.g., melanisation). Larvae did not respond to a therapeutic dose of voriconazole in mixed *Aspergillus* infection [62]. Haemofungin is a novel synthetic drug-like molecule which causes fungal cell swelling and lysis by inhibiting ferrochelatase, the last enzyme in the haem biosynthetic pathway, and is active at low concentrations against pathogenic moulds and yeast. In larvae, haemofungin is non-toxic up to 22.7 mg/kg. Most interestingly, a concentration of 5.7 mg/kg haemofungin improved larval survival to *A. fumigatus* comparable to that of amphotericin (2 mg/kg) [63].

*G. mellonella* larvae have been used to assess the virulence of amphotericin B resistant and susceptible isolates of *Aspergillus tereus* and to assess the potential of amphotericin B for the control of infection. The results demonstrated that amphotericin B was active against the fungus but also stimulated the larval immune response [35]. *G. mellonella* larvae have been utilized to assess the virulence potential of a range of mucormycetes (e.g., *Rhizopus* spp., *Rhizomucor* spp., *Mucor* spp.) and demonstrated the virulence potential was strain and infection dose specific. In addition, the ability of the fungus to tolerate oxidative stress was also a critical factor in its ability to cause disease [10]. The in vivo antifungal potential of liposomal amphotericin B, posaconazole, isavuconazole and nystatin against the fungal pathogens was assessed in larvae. Good control of infection was achieved with nystatin and posaconazole, but not with liposomal amphotericin B and isavuconazole [10]. *Madurella mycetomatis* infection results in the formation of large subcutaneous lesions in humans and antifungal therapy is difficult due to the formation of grains within infected tissue. *G. mellonella* larvae are susceptible to infection by *M. mycetomatis* and infected tissue also shows the presence of grains. Larvae have been used to identify potential antifungal therapies for use in humans and highlighted that amphotericin B and terbinafine prolonged larval survival while azoles proved ineffective [43]. A recent study screened 800 compounds and tested the ten most active compounds for antifungal efficacy in larvae against *M. mycetomatis*. Several compounds enhanced survival and/or reduced fungal burden. Fenarimol analogues, especially EPL-BS0178 appeared most potent possibly due to their polarity, permeability and tissue distribution allowing penetration of *M. mycetomatis* grains in vivo [47].

White nose syndrome is a fungal disease of bats caused by *Pseudogymnoascus destructans* and this has been responsible for the deaths of millions of bats in North America in recent years. *G. mellonella* larvae were shown to be susceptible to disease by *P. destructans* and were used to demonstrate that trifluoperazine and amphotericin B could be used to prevent infection and thus highlighted potential treatment options for use in bats [64].

*G. mellonella* larvae have been adapted to study the virulence of Fusarium spp and to investigate antifungal therapy and have generated results similar to those obtained from murine studies. Larval killing was dependent on temperature (more rapid at 30 °C compared to 37 °C), strain, inoculum and conidia morphology [65]. *Mucor circinelloides* is an opportunistic fungal pathogen that commonly infects patients with an aberrant immune status and presents as nasal, facial or subcutaneous necrosis and disseminated disease [66]. Bastidas et al. 2012 found that the immunosuppressive agent rapamycin, possessed antifungal activity mediated by interactions with FKBP12 and a Tor homolog. *G. mellonella* larvae infected with a lethal dose of *M. circinelloides* R7B were treated with rapamycin (33 mg/kg body weight) and resulted in a 50% survival rate as compared to the 0% control after 5 days [67].

*Trichosporon* species are opportunistic anamorphous basidiomycetes, which cause infections ranging from superficial to systemic in the immunocompromised host and are estimated to be in the top three most common non-*Candida* yeast infection that cause invasive disease in haematological cancer patients [68,69]. *Trichosporon asahii*, *T. asteroides* and *T. inkin* all infected *G. mellonella* larvae and immunosuppressed mice with species-specific differences. However, significant differences were observed between *T. asteroids* species in mice. Both fluconazole and voriconazole improved survival in *G. mellonella* and mice amongst the three species examined. However, amphotericin B improved survival in mice but not in larvae [70].

*G. mellonella* larvae have also been employed to study infection by *Paracoccidioides brasiliensis* and *Paracoccidioides lutzii* the main causative agents of paracoccidioidomycosis, an endemic mycosis in South and Central America. Typically, murine studies can take up to 60 days for infection/therapy, with patient treatment lasting up to 18 months, however, results obtained from larvae infected with *Paracoccidioides* were recorded within 7 days. Larvae were protected from *Paracoccidioides* infection following amphotericin B or itraconazole at therapeutic doses, which decreased fungal burden in larval tissue [71].

## 4. Conclusions

Larvae of *G. mellonella* have become popular models for examining the in vivo toxicity and efficacy of antifungal drugs and give a good indication of the likely dose and effect in mammals without the need to use large numbers of mammals in the initial screens. The use of *G. mellonella* larvae can be extended to study disease development in vivo and to monitor the effects of antifungal agents on specific aspects of infection. Larvae are easy to use, inexpensive to purchase, and are free from the legal/ethical restriction that are applied to the use of mammals. The use of *G. mellonella* larvae in this capacity will never completely remove the need to use mammals in this role, but judicious use of larvae can accelerate the identification of potential therapeutic doses for use in mammals and allow the rapid identification of novel antifungal agents prior to use in mammals. 

## Figures and Tables

**Table 1:** Selected examples of utilization of *G. mellonella* larvae to assess fungal virulence.

Reference	Fungal Virulence and Infection
Brennan et al. 2002 [22]	The virulence of *Candida albicans* mutants correlates between mice and *Galleria mellonella* larvae
Velagapudi et al. 2009 [28]	*Cryptococcus neoformans* virulence is similar in mice and *Galleria mellonella* larvae
Cotter et al. 2002 [21]	Using insects for assessing pathogenicity of yeasts
Mesa-Arango et al. 2013 [31]	*Galleria mellonella* to study the virulence of the *Candida tropicalis* and determine antifungal drug efficacy
Borman et al. 2016 [32]	Virulence of *Candida auris* and other *Candida* spp. In *Galleria mellonella*
Reeves et al. 2004 [24]	Gliotoxin Production and Virulence of *Aspergillus fumigatus* in *Galleria mellonella*
Slater et al. 2011 [23]	Pathogenicity of *Aspergillus fumigatus* mutants in *Galleria mellonella* matches that in mice
St. Leger et al. 2000 [33]	*Aspergillus flavus* pathogenicity in *Galleria mellonella*
Navarro-Velasco et al. 2011 [34]	*Galleria mellonella* to study *Fusarium oxysporum* mutants.
Maurer et al. 2015 [35]	*Galleria mellonella* to assess infection, virulence and and amphotericin B resistance of *Aspergillus terreus*
Thomaz et al. 2013 [36]	*Galleria mellonella* to study *Paracoccidioides lutzii* and *Histoplasma capsulatum*

**Table 2:** Examples of use of *G. mellonella* larvae with antifungal agents.

Assessment of Antifungal Activity	
Rowan et al. 2009 [51]	Use of *Galleria mellonella* larvae to evaluate the in vivo antifungal activity of [Ag_2_(mal)(phen)_3_]
Fuchs et al. 2016 [52]	Micafungin elicits an immunomodulatory Effect in *Galleria mellonella* and mice
Ames et al. 2017 [53]	*Galleria mellonella* as a host model to study *Candida glabrata* virulence and antifungal efficacy
Aneja et al. 2016 [48]	Effect of novel triazole-amino acid hybrids on growth and virulence of *Candida* species: in vitro and in vivo studies.
Kelly et al. 2011 [44]	Caspofungin primes the immune response of the larvae of *Galleria mellonella* and induces a non-specific antimicrobial response
